# *PLP1-lacZ* transgenic mice reveal that splice variants containing “human-specific” exons are relatively minor in comparison to the archetypal transcript and that an upstream regulatory element bolsters expression during early postnatal brain development

**DOI:** 10.3389/fncel.2022.1087145

**Published:** 2023-01-11

**Authors:** Pankaj Patyal, Daniel Fil, Hamdan Hamdan, Patricia A. Wight

**Affiliations:** Department of Physiology and Cell Biology, University of Arkansas for Medical Sciences, Little Rock, AR, United States

**Keywords:** myelin proteolipid protein gene (*PLP1*), splice variants, gene regulation, brain, RT-qPCR, transgenic mice, *lacZ* reporter gene

## Abstract

Much of what is known about the mechanisms that control the developmental expression of the myelin proteolipid protein gene (*PLP1*) has been attained through use of transgenic animal models. In this study, we analyzed expression of related transgenes which utilize *PLP1* genomic DNA from either human or mouse to drive expression of a *lacZ* reporter. Human *PLP1* (*hPLP1*) sequence span either the proximal 6.2 or 2.7 kb of 5′-flanking DNA to an internal site in Exon 2, while those from mouse comprise the proximal 2.3 kb of 5′-flanking DNA to an analogous site in Exon 2. Transgenes with *hPLP1* sequence were named, in part, to the amount of upstream sequence they have [6.2hPLP(+)Z/FL and 2.7hPLP(+)Z]. The transgene containing mouse sequence is referred to here as mPLP(+)Z, to denote the species origin of *PLP1* DNA. Mice which harbor the 6.2hPLP(+)Z/FL transgene were used as a model system to investigate the developmental expression of splice variants that incorporate supplementary exons from what is classically defined as *PLP1* intron 1. While expression of the splice variants were detected in brain through RT-PCR analysis, they are present at much lower levels relative to the archetypal (classic) transcript. Additionally, we show that mice which harbor the 6.2hPLP(+)Z/FL transgene demonstrate wide-ranging expression throughout brain at P2, whereas expression of mPLP(+)Z is quite limited at this age. Therefore, we generated new transgenic mouse lines with the 2.7hPLP(+)Z transgene, which contains *hPLP1* sequence orthologous to just that in mPLP(+)Z. Of the seven lines analyzed, six showed higher levels of 2.7hPLP(+)Z expression in brain at P21 compared to P2; the other line expressed the transgene, only weakly, at either age. This trend, coupled with the robust expression observed for 6.2hPLP(+)Z/FL at P2, suggests that the distal 3.5 kb of 5′-flanking *PLP1* DNA specific to 6.2hPLP(+)Z/FL contains regulatory element(s) important for promoting early postnatal expression in brain.

## Introduction

The essential role played by the myelin proteolipid protein (PLP) in normal functioning of the central nervous system (CNS) is illustrated by the fact that mutations in *PLP1* result in either Pelizaeus-Merzbacher disease (PMD) or spastic paraplegia type 2 (SPG2), which are X-linked leukodystrophies (Wolf et al., 1999; Inoue, 2005; Garbern, 2007). In addition to oligodendrocytes, which are the main producers of PLP, other cell types are involved in the pathobiology of these *PLP1*-related disorders including neurons, astrocytes, and microglia (Laukka et al., 2016; Sarret et al., 2016). Symptoms of PMD include ataxia, spasticity, delays in reaching developmental milestones, loss of motor abilities, and the progressive deterioration of intellectual function (Wolf et al., 1999). Life span is shortened in these patients. SPG2 is characterized primarily by spastic gait and autonomic dysfunction (Wolf et al., 1999). When additional CNS signs are present (e.g., ataxia), it is referred to as complicated SPG2. Only patients with uncomplicated SPG2 have a normal life expectancy; patients with complicated SPG2 typically succumb between their fourth and seventh decade (Wolf et al., 1999). *PLP1* mutations that cause these disorders include duplications and deletions, suggesting that *PLP1* expression is akin to a Goldilocks scenario–normal function requires that the amount of protein produced must be just right.

The general structure of *PLP1* is quite similar in human and mouse, with seven major exons distributed over nearly 16 kb of DNA. Two major isoforms are generated due to alternative splicing of Exon 3 sequence; PLP, a 276 amino acid polypeptide, and a similar protein called DM20, which lacks PLP residues 116–150 (Nave et al., 1987; Simons et al., 1987). PLP constitutes the most abundant protein present in CNS myelin (Jahn et al., 2009, 2020). Both PLP and DM20 are highly conserved in mammals; there is 100% identity between the isoforms in human and mouse.

**Figure 1:**
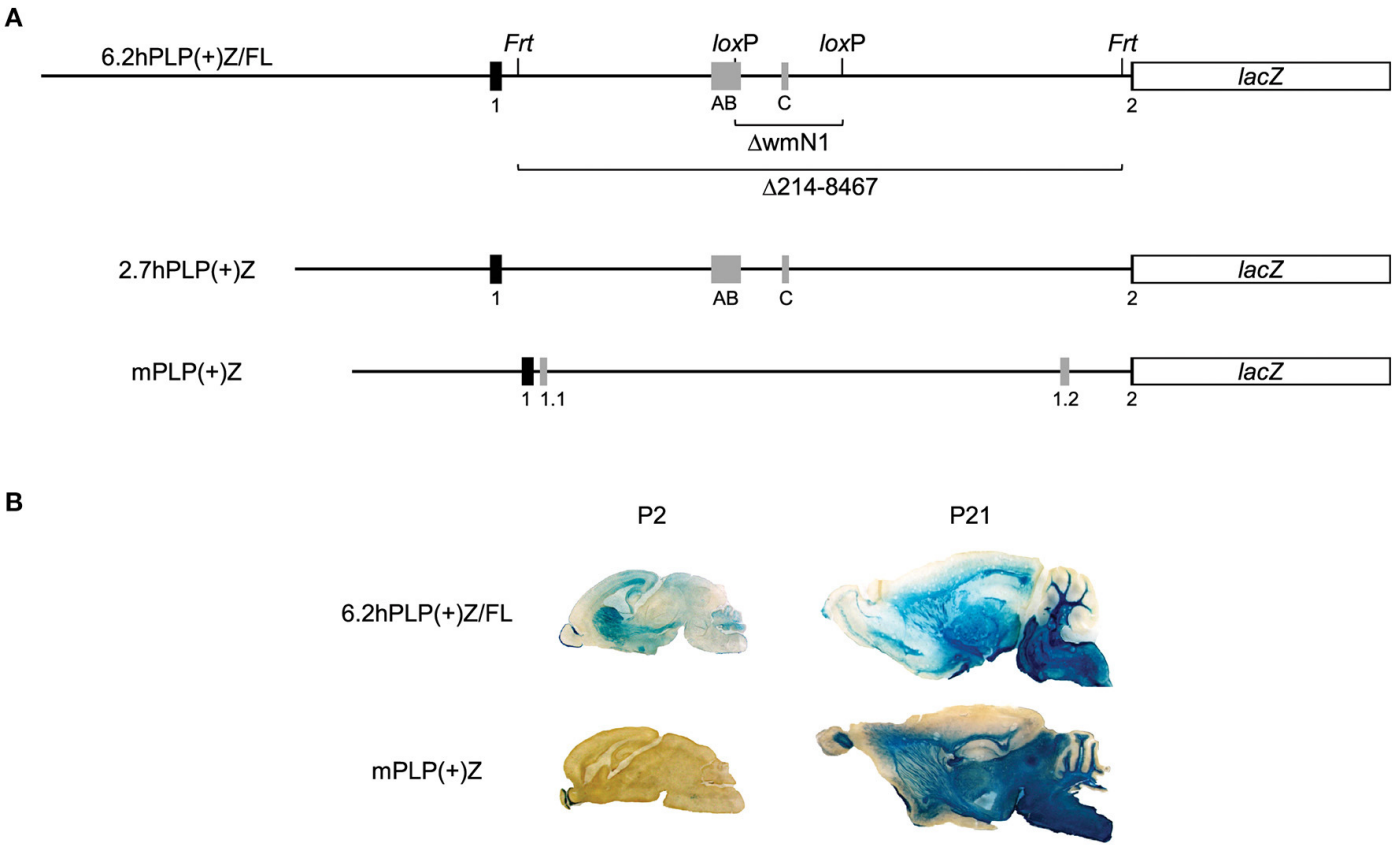
Expression of the 6.2hPLP(+)Z/FL transgene in brain is wide-spread at P2, unlike mPLP(+)Z. Schematic of the 6.2hPLP(+)Z/FL, 2.7hPLP(+)Z, and mPLP(+)Z transgenes **(A)**. The 6.2hPLP(+)Z/FL transgene utilizes *hPLP1* DNA spanning the proximal 6.2 kb of 5′-flanking DNA to first 38 bp of Exon 2 to drive expression of a *lacZ* reporter cassette. Black boxes indicate *hPLP1* Exon 1 and the beginning of Exon 2, while gray boxes depict supplementary Exons AB and C. Black lines indicate *PLP1* 5′-flanking or intron 1 DNA. A pair each of *Frt* and *lox*P sites were incorporated in 6.2hPLP(+)Z/FL for downstream removal of wmN1 enhancer region (6.2hPLPΔwmN1) and most the *hPLP1* intron 1 (6.2hPLPΔ214-8467). The 2.7hPLP(+)Z transgene is similar to 6.2hPLP(+)Z, except it contains only 2.7 kb of 5′-flanking *hPLP1* DNA and does not have any recombination sites engineered into intron 1. The 2.7hPLP(+)Z construct includes only *PLP1* sequences orthologous to those present in mPLP(+)Z. The mPLP(+)Z transgene contains 2.3 kb of 5′-flanking *mPlp1* DNA to an analogous site within the beginning of Exon 2, which is used to drive the same *lacZ* expression cassette. *mPlp1* Exon 1 and the beginning of Exon 2 are indicated by black boxes, while supplementary Exons 1.1 and 1.2 are represented by gray boxes. Relative expression of 6.2hPLP(+)Z/FL and mPLP(+)Z in brain as shown by staining with X-gal **(B)**. Mid-sagittal slices (30 μm) of brain were obtained from 6.2hPLP(+)Z/FL (Line 777) and mPLP(+)Z (Line26H) mice at the indicated ages. Tissue sections from P2 mice were incubated in X-gal stain for 6 h, whereas sections obtained from P21 mice were incubated for only 1 h. While both transgenes exhibited robust staining with X-gal in white matter areas of brain at P21, expression of mPLP(+)Z was restricted primarily to the olfactory bulb at P2, whereas expression of 6.2hPLP(+)Z/FL transgenic mice was much broader at this age.

In addition to the classic *PLP/DM20* transcripts (generated in all mammals), other splice variants are produced in some species through addition of supplementary exons. As depicted in Figure 1A, both human and mouse *PLP* intron 1 DNA contains two (unrelated) supplementary exons. In human, Exon AB and Exon C are incorporated independently of one another in the fetal and adult CNS (Sarret et al., 2010). These supplementary exons are largely restricted to the human species, although Exon AB or A-specific portions of it have been found in transcripts from macaque, while Exon C-containing transcripts are present in bovine (Sarret et al., 2010). These novel splice variants are expressed preferentially in neurons (Sarret et al., 2010), unlike their classic (*PLP/DM20*) counterparts, which are better expressed in oligodendrocytes. Splice variants that incorporate only A-specific portions (A and A′) of Exon AB encode proteins with nine extra amino acids at the N-terminus of PLP or DM20. These isoforms get transported to the plasma membrane as shown by transfection analysis with applicable expression constructs (Sarret et al., 2010). Thus, these novel proteins could pose as targets for immune-mediated degeneration of axons/neurons in persons with multiple sclerosis. In contrast, it is not known whether splice variants that contain Exon AB in its entirety, or Exon C, are translated into protein, or function solely as non-coding RNAs. If they do encode a protein product, the start of translation is predicted to be near the end of *hPLP1* Exon 4, due to multiple in-frame stop codons downstream of the traditional start site in Exon 1 that is used to make the classic (PLP/DM20) products. This in turn would yield a peptide corresponding to the last 72 amino acids of PLP (Sarret et al., 2010). Intriguingly, a secreted C-terminal PLP product has been shown to increase proliferation of oligodendrocyte lineage cells, *in vitro* (Yamada et al., 1999). Thus, it is possible that Exon AB- or C-containing transcripts may encode a protein that acts as a growth factor.

Exons AB and C are not utilized as such in mouse even though the orthologous sequences are moderately conserved (51 and 77% identity, respectively), compared to human. This is not the result of insufficient quantities of human-specific splicing components since an earlier study (Hamdan et al., 2015) showed that all of the “human-specific” splice variants are generated in a mouse cell line transfected with *hPLP1-lacZ* constructs. Therefore, lack of “human-specific” transcripts in *PLP1*-null patients may have unique consequences in man.

The *PLP1* gene in mouse (*mPlp1*) contains a different pair of supplementary exons in intron 1; Exon 1.1 (Bongarzone et al., 1999) and Exon 1.2 (Li et al., 2009), as illustrated in Figure 1A. Exon 1.1-containing splice variants encode an isoform with an additional 12 amino acid to the classic product and this leader protein retained within the cell soma (Bongarzone et al., 1999).

To elucidate the mechanisms regulating *PLP1* expression, our group has generated multiple transgenic mouse lines which use the first half of the *PLP1* gene from human or mouse to drive expression of a *lacZ* reporter cassette. The 6.2hPLP(+)Z/FL transgene (Hamdan et al., 2018) contains *PLP1* derived from human that spans the proximal 6.2 kb of 5′-flanking DNA to the first 38 bp of Exon 2. We have used 6.2hPLP(+)Z/FL mice as a model system to study the developmental expression of the “human-specific” splice variants, which incorporate a supplementary exon (or a portion thereof) from what it classically defined as *PLP1* intron 1 DNA. As demonstrated here, by RT-qPCR analysis, expression of these splice variants is far less than the classically spliced transcripts. Curiously, transgenic lines harboring 6.2hPLP(+)Z/FL demonstrate substantial expression in brain at postnatal day 2 (P2) (Hamdan et al., 2018), whereas lines with either mPLP(+)Z (Wight et al., 1993) or mPLP(+)/FL (Patyal et al., 2020) have very limited expression at this age. However, expression is wide-spread in brain at P21 for all of the lines, regardless of the species origin of *PLP1* DNA. Because 6.2hPLP(+)Z/FL contains more *PLP1* 5′-flanking DNA than does mPLP(+)Z or mPLP(+)Z/FL, we generated additional transgenic mice with the 2.7hPLP(+)Z transgene, which just contains *hPLP1* sequence orthologous to that present in mPLP(+)Z and mPLP(+)Z/FL, to determine whether the upstream sequence selective to 6.2hPLP(+)Z/FL may account for the considerable expression observed at P2. Here we demonstrate that 2.7hPLP(+)Z expression in brain is higher at P21 than P2, suggesting that a transcription regulatory element located 2.7 to 6.2 kb upstream of *hPLP1* Exon 1, is important for governing expression during early postnatal brain development.

## Materials and methods

### *PLP1-lacZ* transgenic mouse lines

Generation of transgenic mouse lines that harbor a *lacZ* reporter cassette driven by either human or mouse *PLP1* sequences (Lines 777 and 26H, respectively), have been described, previously (Wight et al., 1993; Hamdan et al., 2018). The 6.2hPLP(+)Z/FL transgene contains human *PLP1* (*hPLP1*) sequence that spans the proximal 6.2 kb of 5′-flanking DNA to the first 38 bp of Exon 2, whereas the PLP(+)Z transgene contains mouse *Plp1* (*mPLP1*) sequence that span the proximal 2.3 kb of 5′-flanking DNA to the first 37 bp of Exon 2. PLP(+)Z is referred to here as mPLP(+)Z, to denote the species origin of *PLP1* DNA. The 6.2hPLP(+)Z/FL transgene also contains a pair of *Frt* sites (F) and a pair of *lox*P sites (L) in *hPLP1* intron 1 for downstream removal of the intervening sequences. Cre recombinase was used to generate 6.2hPLPΔwmN1 mice, which are missing *hPLP1* intron 1 positions 3174-4660, but maintain the same integration site as does the parental (unrearranged) line (Hamdan et al., 2018).

To generate 6.2hPLPΔ214-8467 mice which lack *hPLP1* intron 1 positions 214-8467, 6.2hPLP(+)Z/FL hemizygous mice from Line 777 were bred with Flp-deleter mice [B6.129S4-*Gt(ROSA)26Sor*^*tm*1(*FLP*)*Dym*^/RainJ, Jackson Laboratory]. The resulting progeny was then backcrossed to the Flp-deleter for 6 generations in order to remove the sequence flanked by *Frt* sites from all copies of the transgene. Presence or absence of the sequence targeted for deletion was determined by PCR analysis using an “internal” primer pair (sense primer: 5′-CGGGGGTTCCATGGTTTAAATGAGTCGT-3′; antisense primer: 5′-AAGGGGAAACAGTCAGGCACATCCAGTA-3′) in conjunction with an “external” primer set (sense: 5′-CCAGCTCTGAAAAGACACTCTTCCCAAG-3′; antisense: 5′-CACCAAAGATGAGCCTCAGAAGCCCAAG-3′). PCR reactions were run in a thermocycler under the following stepwise conditions: 94°C for 4 min; 35 repetitive cycles of 94°C for 75 s (denaturation), 60°C for 75 s (annealing), and 72°C for 90 s (extension); and a final step of 72°C for 7 min. PCR products were analyzed by gel electrophoresis. A 267-bp amplicon is generated with the external primer pair if the sequence targeted for deletion has been removed. A 211-bp amplicon is produced from the internal primer pair if the targeted sequence is still present.

Mice that contain the 2.7hPLP(+)Z transgene were generated by the Transgenic Animal Model Core at the University of Michigan, Ann Arbor through microinjection of a 17.9-kb XhoI-NotI fragment from the 2.7hPLP(+)Z plasmid (Hamdan et al., 2015) into fertilized C57BL/6 mouse eggs. The transgene contains *hPLP1* sequences that span the proximal 2.7 kb of 5′-flanking DNA to the first 38 bp of Exon 2, which are used to drive expression of the same *lacZ* reporter cassette found in 6.2hPLP(+)Z/FL and mPLP(+)Z. The *hPLP1* DNA in 2.7hPLP(+)Z is wholly orthologous to that present in mPLP(+)Z.

Mice that harbor a given *PLP1-lacZ* transgene were identified through PCR analysis of genomic DNA isolated from tail biopsies according to the methods of Truett et al. (2000), in conjunction with the *lacZ* primer pair described by Stratman et al. (2003). The conditions for PCR were the same as those described above except that only 30 cycles of amplification were applied. Breeding colonies of the transgenic mouse lines were maintained in the hemizygous state in order to generate non-transgenic (wild-type; WT) littermates as controls for some experiments. Experimental groups were comprised of both male and female mice at a given postnatal day (P) of age. All procedure involving the use of mice were approved by the Institutional Animal Care and Use Committee at the University of Arkansas for Medical Sciences in compliance with the Public Health Service Policy on Humane Care and Use of Laboratory Animals and the National Research Council's Guide for the Care and Use of Laboratory Animals, and adhered to ARRIVE guidelines (Kilkenny et al., 2010).

### Conventional reverse transcription-PCR

6.2hPLP(+)Z/FL mice (Line 777) at P2 of age and their non-transgenic (WT) littermates were first anesthetized by indirect placement on ice for 15–20 min and then euthanized by decapitation using a sharp pair of scissors. Immediately afterwards, the brain was dissected and total RNA extracted using the RNeasy Mini Kit (Qiagen, Valencia, CA) according to the manufacturer's directions. The concentration of RNA was determined using a NanoDrop 2000c Spectrophotometer (Thermo Scientific, Wilmington, DE). First strand cDNA synthesis was performed with the iScript gDNA Clear cDNA Synthesis Kit (Bio-Rad, Hercules, CA) per the supplier's instructions using 1 μg of total RNA in a final reaction volume of 20 μl. Afterwards, the mixture was stored at −70°C until needed or used immediately as template DNA for PCR.

PCR was performed with 2 μl of the cDNA solution using the JumpStart REDTaq ReadyMix PCR Reaction Mix (Sigma-Aldrich, St. Louis, MO) according to the manufacturer's guidelines. Amplicons derived from the archetypal (classic, Cl) *hPLP1-lacZ* transcript or related splice variants [which contain Exon AB in its entirety or a portion of it (A or A′) or, alternatively, Exon C] were generated using various *hPLP1* sense (forward, F) primers and one of two common antisense (reverse, R) primers aimed at the *lacZ* expression cassette. The *hPLP1* sense primers were as follows: 5′-CTGAACAAAGTCAGCCACAAAG-3′ (Exon 1-F); 5′-CTGGGTACAGGTCTTTGTCTTG-3′ (Exon A-F1); 5′-GACAGTGACATGATCAGGAAGG-3′ (Exon A′-F); 5′-GAGTCCCAAGGAAGAGAATAAGG-3′ (Exon AB-F); 5′-TTCGGCCTTGTCAACTACTG-3′ (Exon C-F). The corresponding antisense primers are as follows: 5′-GTTGAAACGCTGGGCAATATC-3′ (LacZ-R1); 5′-GAGGTGCTGTTTCTGGTCTT-3′ (LacZ-R2). The reaction was run in a thermocycler under the following stepwise conditions: 94°C for 4 min; 40 repetitive cycles of 94°C for 75 s (denaturation), 60°C for 75 s (annealing), and 72°C for 90 s (extension); and a final step of 72°C for 7 min. PCR products were fractionated on a 1% agarose gel in TAE buffer (40 mM Tris pH 8.0, 20 mM Acetate, and 1 mM EDTA). Images of gels were captured on a ChemiDoc MP Imaging System (Bio-Rad). Bands of the various amplicons were cut out of the gel and eluted using the QIAquick Gel Extraction Kit system (Qiagen, Valencia, CA) according to the manufacturer's instructions. The identity of the amplicons was validated by sequencing the eluted DNA using the same primers by which a particular amplicon was generated, with help from the UAMS DNA Sequencing Core Facility.

### Reverse transcription-quantitative PCR analysis

*PLP1-lacZ* transgenic mice at various ages were anesthetized deeply with isoflurane (or by laying young animals, indirectly, on ice for 15–20 min) and then decapitated using a sharp pair of scissors. Immediately afterwards, the brain was harvested, total RNA extracted, and first strand cDNA synthesis performed, as described above, using 1 μg of total RNA in a final reaction volume of 20 μl. The cDNA reaction mixture was diluted by adding either 70 μl [6.2hPLP(+)Z/FL] or 180 μl [2.7hPLP(+)Z] of Nuclease-Free Water (Life Technologies, Carlsbad, CA) and stored at −20°C until use as template in qPCR.

Real-time PCR was performed using either 4.5 μl [6.2hPLP(+)Z/FL] or 2 μl [2.7hPLP(+)Z] of the diluted cDNA mixture along with the TaqMan Fast Advanced Master Mix (Applied Biosystems, Foster City, CA) and a particular TaqMan Gene Expression Assay (Applied Biosystems) in a final volume of 10 μl. The reaction was performed under the supplier's recommended conditions in a StepOnePlus Real-Time PCR System (Applied Biosystems). All custom TaqMan Gene Assays contained a common reverse primer (LacZ-R1), whereas different forward primers and probes were included in order to detect a given splice species expressed in 6.2hPLP(+)Z/FL mice. Archetypal (classic) transcripts were detected using Exon 1-F as the forward primer with a custom probe (5′-ACATGGGCTTGT-3′). The following forward primers and corresponding probes were included in the custom TaqMan Gene Assays to detect splice variants that incorporate *hPLP1* Exon A (Exon A-F2 primer: 5′-ATCCCCTGGGTACAGGTCTT-3′; custom probe: 5′-TAGAAATGCCTTCCCTGCCCACAG-3′), Exon A′ (Exon A′-F primer; custom probe: 5′-AGAGTGCTGTGCAAGATGTCTGGT-3′), Exon AB (Exon AB-F primer; custom probe: 5′-AGGAGA GAGGAGGAAATGGTGGGA-3′) or Exon C (Exon C-F primer; custom probe: 5′-AGAGTG CTGTGCAAGATGTCTGGT-3′). In addition, commercially available primer/probe sets from Applied Biosystems for analysis of the housekeeping genes, *18S* (Mm03928990_g1, Cat# 4448489) and *Gusb* (Mm01197698_m1, Cat# 4331182) were also used. PCR reactions were run in duplicate for each biologic (cDNA) sample with a given primer/probe set. The relative level of gene expression was calculated using the 2^−ΔΔCt^ method. Results are presented as the mean level ± *SD* (n = 3) relative to that from the *18S* and *Gusb* reference genes. Expression of just the classic transcript was assessed with 2.7hPLP(+)Z mice.

### β-galactosidase histochemistry

To determine the areas in brain that express the *PLP1-lacZ* transgenes, mice at P2 or P21 of age were anesthetized as described in the preceding section, and perfused intracardially with cold phosphate-buffered saline (PBS) pH 7.3, followed by fixative (1.0% glutaraldehyde in 0.2 M PBS, pH 7.3), and then fixative with 10% sucrose. The perfused animals were left undisturbed for 1 h. Subsequently, brains were harvested and immersed in fixative containing 10% sucrose. After sinking, the brains were transferred to the same fixative containing 25% sucrose (overnight at 4°C), covered with Tissue-Plus O.C.T. Compound (Fisher Health Care, Houston, TX), and stored at −70°C until cryostat sectioning. Brain slices (20 μm) were incubated with the chromogenic substrate 5-bromo-4-chloro-3-indolyl-β-D-galactopyranoside (X-gal; Research Organics, Cleveland, OH) for ≤1 h (P21) or 6 h (P2), as previously described (Wight et al., 1993). Images were captured under light microscopy.

### β-galactosidase enzyme assay

*PLP1-lacZ* transgenic mice and their non-transgenic (WT) littermates at P2 or P21 of age were anesthetized and then decapitated using a sharp pair of scissors as described above. Brains were rapidly dissected and homogenized separately in Lysis Solution [0.2% Triton X-100 and freshly added phenylmethylsulfonyl fluoride (0.2 mM) and leupeptin (5 μg/ml) in 100 mM potassium phosphate, pH 7.8]. Homogenates were centrifuged at 12,500 g for 10 min at 4°C. The resulting supernatants were incubated at 48°C for 1 h to inactivate any endogenous β-galactosidase (β-gal) activity according to the methods of Young et al. (1993). The lysates were centrifuged at 12,500 g for 5 min at 4°C, and the resulting supernatants (10 μl) assayed for β-gal activity in triplicate using the Galacto-Light Plus Kit as previously described (Pereira et al., 2013). Lysate protein concentrations were determined with the Pierce BCA Protein Assay Kit (Thermo Scientific, Rockport, IL). Results are presented as the mean ± *SD* of β-gal activity (relative light units; RLU) per μg of total protein from three or more animals for each transgenic line/genotype.

## Results

### *PLP1-lacZ* transgene activity is extensive in early postnatal mouse brain with 6.2hPLP(+)Z/FL, but not mPLP(+)Z

To ascertain the mechanisms that regulate *PLP1* gene expression, our group has generated multiple transgenic mouse lines which utilize *PLP1* genomic DNA to drive expression of a *lacZ* reporter cassette. The 6.2hPLP(+)Z/FL transgene (Hamdan et al., 2018) contains *PLP1* sequence derived from human which span the proximal 6.2 kb of 5′-flanking DNA to an internal site in Exon 2, while the PLP(+)Z transgene (Wight et al., 1993), designated here as mPLP(+)Z, contains sequence derived from mouse that encompasses the proximal 2.3 kb of 5′-flanking DNA to an analogous site in Exon 2 (Figure 1A). Pairs of *Frt* and *lox*P sites (each) were incorporated in *PLP1* intron 1 of the 6.2hPLP(+)Z/FL transgene, for downstream removal of the intervening sequences. As shown by staining with X-gal, both the 6.2hPLP(+)Z/FL (Line 777) and mPLP(+)Z (Line 26H) transgenes exhibit substantial expression in white matter areas of brain from mice at P21 of age (Figure 1B). However, at P2, expression of mPLP(+)Z was limited primarily to the olfactory bulb, whereas 6.2hPLP(+)Z/FL expression was far more widespread as evidenced by staining in olfactory bulb, neostriatum, corpus callosum, subventricular zone, and the developing cerebellum (Figure 1B). These results are not peculiar to the particular lines examined here as similar trends were observed with other lines (Wight et al., 1993; Hamdan et al., 2018; Patyal et al., 2020). Thus, there seems to be an inherent difference between expression of 6.2hPLP(+)Z/FL and mPLP(+)Z in early postnatal mouse brain.

**Figure 2:**
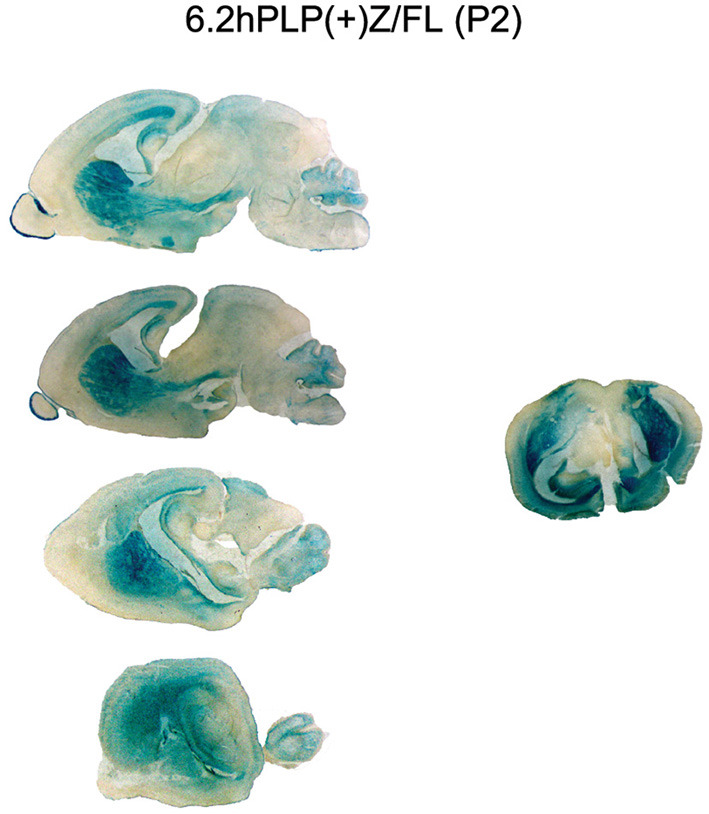
Expression of the 6.2hPLP(+)Z/FL transgene is extensive throughout brain at P2 as demonstrated by staining with X-gal. Mouse brain sections (30 μm) obtained from 6.2hPLP(+)Z/FL mice (Line 777) were stained with X-gal for 6 h. A panel of sagittal tissue slices medial to lateral (top to bottom) from a single mouse is shown on the **left** side. A mid-coronal section from another mouse is shown on the **right** side.

To get a better understanding of exactly where the 6.2hPLP(+)Z/FL transgene is being expressed in brain at P2, additional staining with X-gal was performed on a panel of sagittal sections obtained from a mouse from Line 777, as well as a coronal section obtained from a littermate. As shown in Figure 2, expression of the 6.2hPLP(+)Z/FL transgene was extensive throughout the brain of mice at P2. In fact, the overall level of 6.2hPLP(+)Z/FL expression is higher at P2 than P21 as determined in β-gal enzyme assays of whole brain homogenates (Table 1). Substantial levels of transgene expression in brain also have been detected at P2 in other 6.2hPLP(+)Z/FL lines (Hamdan et al., 2018), suggesting that this is a common finding.

**Table 1:** β-gal activity in brain of *hPLP1-lacZ* mice^*^.

**Genotype**	**P2**	**P21**
6.2hPLP(+)Z/FL	7,684 ± 553	4,015 ± 21
WT	142 ± 11	134 ± 2
6.2hPLPΔwmN1	306 ± 7	206 ± 12
WT	109 ± 3	128 ± 1
6.2hPLPΔ214-8467	155 ± 6	178 ± 9
WT	130 ± 16	152 ± 5

^*^β-gal activity is reported as the mean RLU/μg of protein ± SD (n ≥ 3) in whole brain homogenates prepared from mice containing either the parental 6.2hPLP(+)Z/FL transgene or rearranged transgenes at the indicated postnatal days (P) of age from Line 777 (Hamdan et al., 2018).

### Human *PLP1* intron 1 positions 214–8467 are required for expression of an *hPLP1-lacZ* transgene in brain

Previously our group has shown that removal of the wmN1 enhancer region and adjoining (upstream) 330 bp of *hPLP1* intron 1 sequences in 6.2hPLP(+)Z/FL greatly diminishes expression of the rearranged (6.2hPLPΔwmN1) transgene in brain (Hamdan et al., 2018). However, 6.2hPLPΔwmN mice still demonstrate a low level of transgene expression in brain as determined through β-gal assays (Table 1; compare amounts of activity between transgenic and wild-type animals, at a given age). As illustrated in Figure 1A, the 6.2hPLPΔwmN transgene retains the A-specific portion of supplementary Exon AB as well as the beginning of the B-specific portion. Thus, it is possible that the residual amount of transgene activity produced by 6.2hPLPΔwmN may, in part, be due to expression of splice variants that selectively contain either A or A′ sequence. To test the effects from complete loss of Exon AB, the majority of *hPLP1* intron 1 was deleted using the same parental line of 6.2hPLP(+)Z/FL mice. The rearranged line was named 6.2hPLPΔ214-8467, and retains only the initial 213 bp and terminal 112 bp of *hPLP1* intron 1. Removal of the majority of *hPLP1* intron 1 DNA resulted a loss of transgene activity for 6.2hPLPΔ214-8467 (Table 1, Supplementary Figure 1). This could be due to loss of A- or A′-specific splice variants if they are preferentially expressed during early brain development. Alternatively, it may be due to loss of an additional regulatory element from the intron. Tuason et al. (2008) identified a conserved region downstream of wmN1, termed wmN2, that behaves as an enhancer in controlled transgenesis assays.

### The levels of splice variants generated by 6.2hPLP(+)Z/FL are low in comparison with the archetypal (classic) transcript

**Figure 3:**
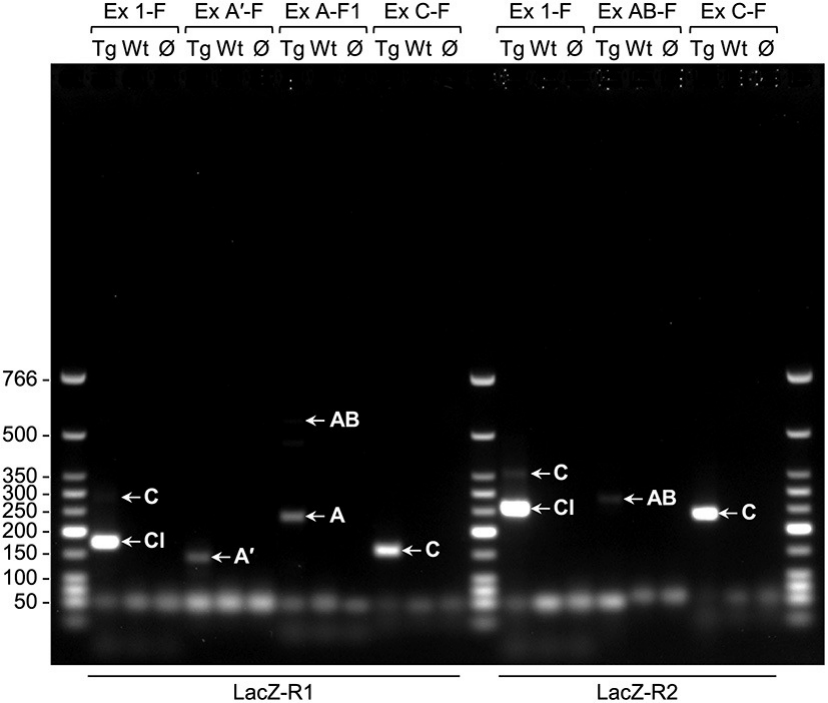
Non-quantitative RT-PCR analysis indicates that the classic (Cl) transcript and splice variants are present in brain of 6.2hPLP(+)Z/FL mice (Line 777) at P2 of age. The primers used in the various PCR reactions are listed above (forward primers) and below (reverse primers) the image of a representative gel (1% agarose). RNA was isolated from transgenic (Tg) or wild-type (Wt) mice and reverse transcribed into DNA; Ø indicates no template DNA. Arrows point to the product(s) formed by a given primer pairs: Exon 1-F/LacZ-R1 (174 bp for the Cl-related amplicon; 282 bp for the C-related amplicon); Exon A′-F/LacZ-R1 (140 bp for the A′-related amplicon); Exon A-F1/LacZ-R1 (237 bp for the A-related amplicon; 553 bp for the AB-related amplicon); Exon C-F/LacZ-R1 (157 bp for the C-related amplicon); Exon 1-F/LacZ-R2 (256 bp for the Cl-related amplicon; 364 bp for the C-related amplicon); Exon AB-F/LacZ-R2 (280 bp for the AB-related amplicon); Exon C-F/LacZ-R2 (239 bp for the C-related amplicon). The wells of the gel are located at the top of the image. Sizes (bp) for the Low Molecular Weight DNA Ladder (New England BioLabs, Ipswich, MA) are indicated to the left of the image.

Because the activity of *lacZ* transgenes is extensive in early postnatal mouse brain when driven by *PLP1* sequences obtained from human, but not mouse, RT-PCR studies were performed to determine whether expression of “human-specific” splice variants could explain the discrepancy. Initially, non-quantitative RT-PCR analysis was performed to determine whether splice variants that incorporate supplementary Exon AB (or just the A or A′ portions) or Exon C are produced in brain of 6.2hPLP(+)Z/FL mice at P2 of age. RNA from non-transgenic (wild-type; WT) littermates was analyzed as well, as a negative control. All of the transgene splice variants (A′, A, AB, and C) were detected through non-quantitative RT-PCR analysis (Figure 3). The identity of the transcripts was confirmed by sequencing of gel eluted DNA fragments.

**Figure 4:**
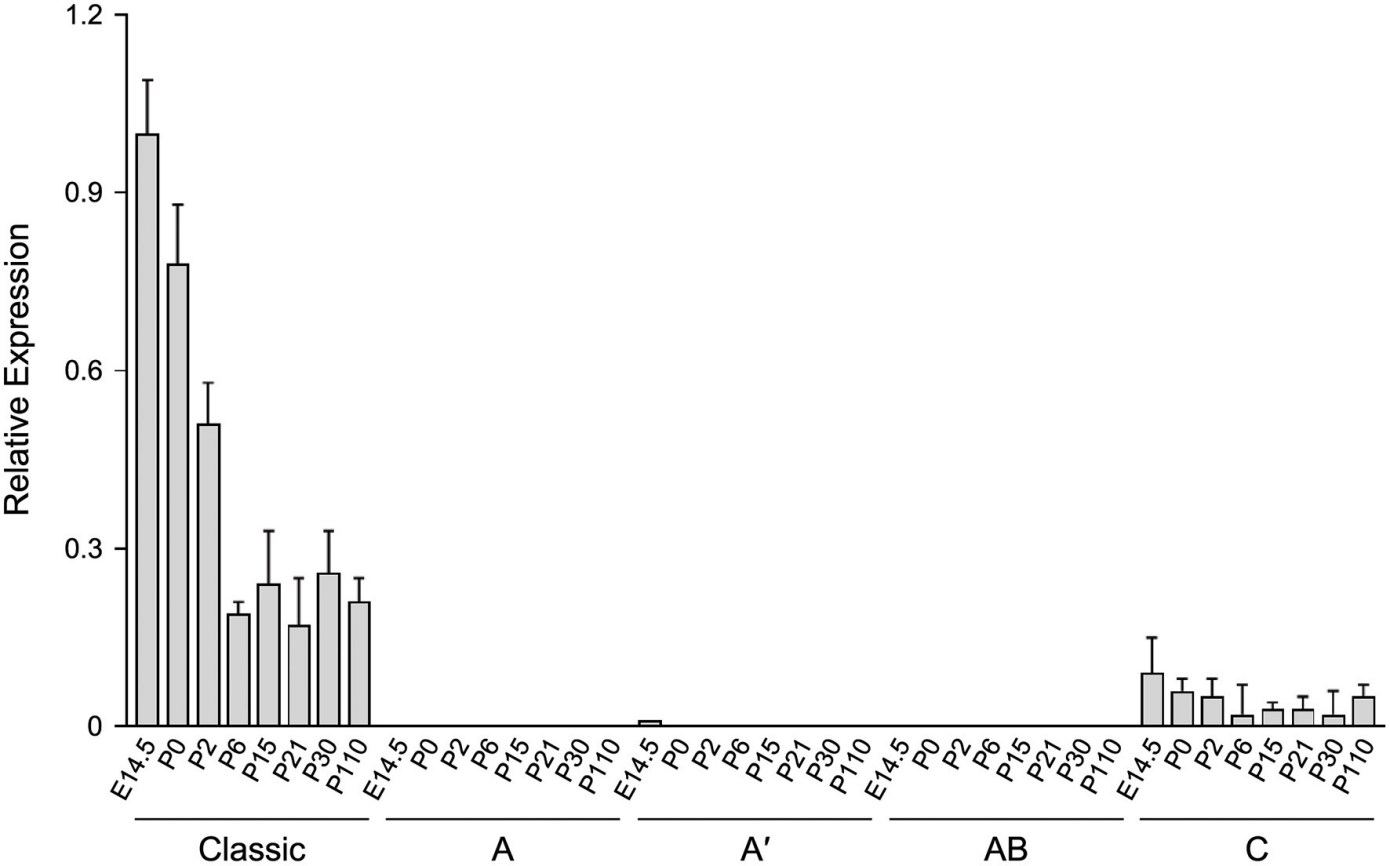
The level of the classic transcript from 6.2hPLP(+)Z/FL is much higher than “human-specific” splice variants, in brain, during development. RT-qPCR analysis was performed using total RNA isolated from brain of mice at the indicated ages. Expression of splice variants containing Exon A, Exon A′ or Exon AB were too low to be detected, except for the Exon A′ splice variant, which was slightly detected at E14.5. A low level of the Exon C-containing variant was detected at all ages, tested. However, the level was less than that of the classic transcript throughout development. Results are reported as the fold mean ± SD (*n* = 3) of the various *hPLP1-lacZ* transcripts, relative to that from the reference genes (*18S* and *GUSb*). Amounts plotted are relative to that generated using a pooled sample of brain RNA from all animals, which was arbitrarily set at one.

To evaluate the developmental profile of the alternative splice variants in comparison to the classic transcript, RT-qPCR analysis was performed with RNA isolated from brain of 6.2hPLP(+)Z/FL mice at different ages (E14.5, P0, P2, P6, P15, P21, P30, and P110). As shown in Figure 4, the splice variant containing Exon C was detected at all ages examined, with the highest level obtained at E14.5. However, the amount of this splice variant was 4–13-fold less than the archetypal (classic) transcript for all ages, examined. Interestingly, the level of the classic transcript was highest at E14.5 and decreased stepwise at P0, P2, and P6 of age and then remained steady out to P110. The relative levels of expression for the other splice variants were extremely low to undetectable in comparison to that of the reference genes (*18S* and *Gusb*). Thus, the early postnatal expression observed with the 6.2hPLP(+)Z/FL transgene does not appear to be due to selective expression of “human-specific” splice isoforms.

### The 2.7hPLP(+)Z transgene is expressed at lower levels in brain of mice at P2 of age compared with P21

**Figure 5:**
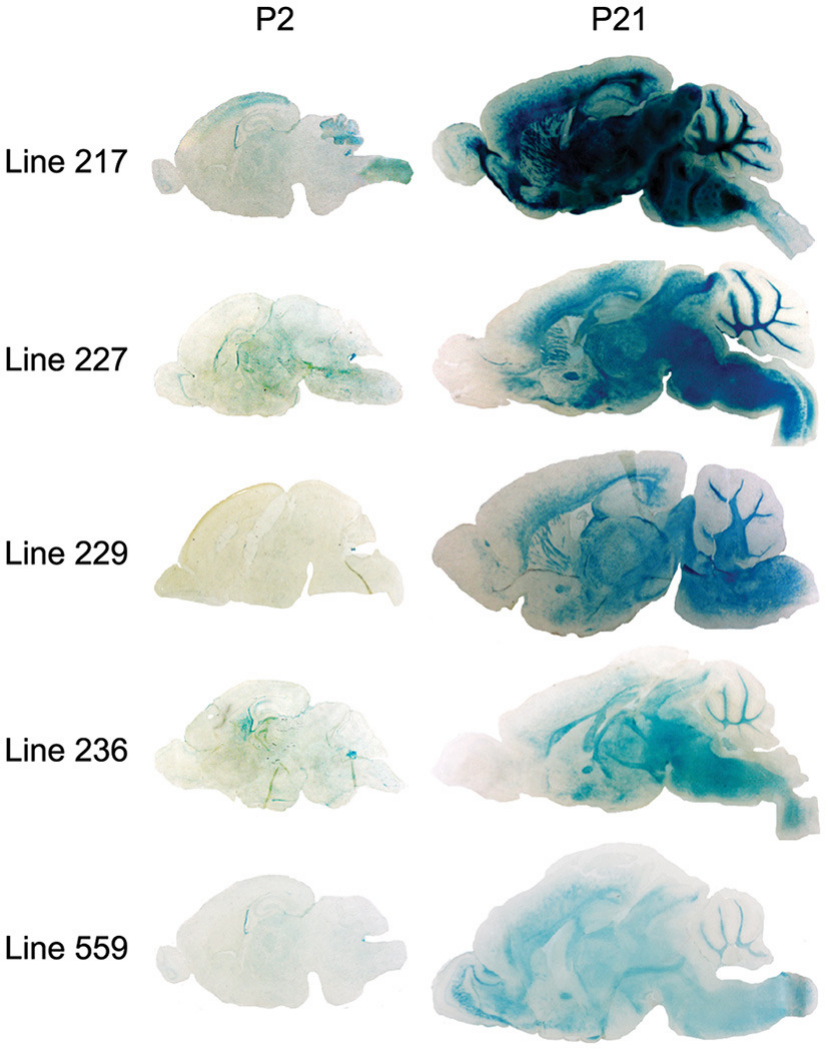
β-gal activity in brain of 2.7hPLP(+)Z transgenic mice lines at P2 and P21 as determined by X-gal staining. Mid-sagittal sections (20 μm) obtained from brains of mice at P2 and P21 of age from Lines 217, 227, 229, 236, and 559 were stained with X-gal; 1 h for tissue slices obtained from P21 animals (except for Line 217, which was stained for only 20 min) and 6 h for sections from P2 mice. Staining was evident in white matter areas of brain in all of the lines at P21, however at P2, there was far less staining.

Because the 6.2hPLP(+)Z/FL transgene contains more 5′-flanking *PLP1* DNA than does mPLP(+)Z (see Figure 1A), it is possible a transcription regulatory element lies within this extra upstream sequence which is important in governing early postnatal expression of the transgene. To investigate this possibility, transgenic mice that harbor the 2.7hPLP(+)Z transgene were generated. The 2.7hPLP(+)Z transgene contains *PLP1* sequences orthologous to that present in the mPLP(+)Z transgene, and has 3.5 kb less of 5′-flanking DNA than does 6.2hPLP(+)Z/FL. It contains 2.7 kb of 5′-flanking *hPLP1* DNA as indicated by its name. Ten (male) founders were generated on the C57BL/6 genetic background out of 43 pups born (23% yield of transgenesis). Transgenic lines were established for nine of the founders; one founder did not show germline transmission. Seven of the lines (Lines 217, 226, 227, 229, 236, 559, and 563) expressed the transgene as determined by significantly higher levels of β-gal activity in homogenates prepared from transgenic mouse brains (P2 and P21 of age) compared to the (background) levels in non-transgenic (WT) littermates (Table 2). The other two lines (Lines 225 and 228) did not express the transgene as determined in β-gal enzyme assays (unpublished data). Lines 217 and 227 were the highest expressing lines, whereas Lines 226 and 559 were low expressing lines (Table 2). Lines 229, 236, and 563 expressed the transgene to intermediate levels. With the exception of Line 226, which is a very low expressing line, expression in the other six lines was higher at P21 than P2 by 1.81–71.55-fold (Table 3). These results are corroborated by X-gal staining of mid-sagittal brain slices obtained from various lines of 2.7hPLP(+)Z mice, which show only weak staining at P2 compared with stronger staining (in white matter areas) at P21, within a given line (Figure 5). While variability in expression between the lines is to be expected due to different sites of chromosomal integration (i.e., position effects) and variable copy number, the trend of lower level of 2.7hPLP(+)Z at P2 compared with P21 remains consistent.

**Table 2:** β-gal activity in brain for 2.7hPLP(+)Z mouse lines^*^.

**Line**	**Genotype**	**P2**	**P21**
217	2.7hPLP(+)Z	26,473 ± 368	71,562 ± 697
	WT	139 ± 18	112 ± 11
226	2.7hPLP(+)Z	392 ± 43	319 ± 35
	WT	84 ± 7	80 ± 3
227	2.7hPLP(+)Z	12,023 ± 168	30,669 ± 1,077
	WT	112 ± 13	113 ± 8
229	2.7hPLP(+)Z	138 ± 26	9,874 ± 19
	WT	122 ± 7	131 ± 3
236	2.7hPLP(+)Z	2,171 ± 271	3,934 ± 262
	WT	132 ± 6	147 ± 17
559	2.7hPLP(+)Z	413 ± 8	928 ± 32
	WT	115 ± 8	110 ± 3
563	2.7hPLP(+)Z	671 ± 14	4,001 ± 561
	WT	145 ± 2	130 ± 7

^*^β-gal activity is reported as the mean RLU/μg of protein ± SD (n ≥ 3) in whole brain homogenates prepared from mice at the indicated postnatal days (P) of age.

**Table 3:** Ratio of transgene RNA and β-gal levels in 2.7hPLP(+)Z mouse brain (P21/P2)^*^.

**Line**	**RT-qPCR**	**β-gal activity**
217	3.53	2.70
226	0.81	0.81
227	1.80	2.55
229	56.63	71.55
236	3.69	1.81
559	2.98	2.25
563	3.07	5.96

^*^RT-qPCR analysis was performed using total RNA isolated from brain of 2.7hPLP(+)Z mice at P2 or P21 of age (n = 3, each). Expression of *lacZ* was determined relative to that for reference genes (*18S* and *Gusb*). Ratios indicate the mean level of transgene RNA or β-gal activity (from Table 2), at P21 over P2.

Relative expression of the transgene in the various lines of 2.7hPLP(+)Z mice was also analyzed by RT-qPCR analysis (Table 3), using RNA isolated from brain of mice at P2 and P21. Levels of transgene (*hPLP1-lacZ*) mRNA in brain were generally higher (1.80–56.63-fold) at P21 than P2 in all of the lines except for Line 226, which is the lowest expressing line. These findings are consistent with those obtained in the β-gal enzyme assays (Table 3). Together, these results suggest that a transcription regulatory element is present in *hPLP1* 5′-flanking DNA, located 2.7–6.2 kb upstream of Exon 1, which is important for governing early postnatal expression in brain.

## Discussion

Transgenic mouse lines that utilize *PLP1* sequences to drive expression of reporter genes have been fundamental tools to uncover the mechanisms which control *PLP1* gene expression during development. In this report, we have made use of several *PLP1-lacZ* lines to expand our knowledge of the central mechanisms regulating *PLP1* gene expression. One of these harbors the 6.2hPLP(+)Z/FL transgene, which contains *hPLP1* sequences that span the proximal 6.2 kb of 5′-flanking DNA to the first 38 bp of Exon 2 (Figure 1A). Because the transgene contains *PLP1* sequences derived from human, it represents the first and only (to the best of our knowledge) animal model system to study the developmental expression of alternative splice variants that incorporate supplementary Exons (AB and C) from *hPLP1* intron 1. These exons are primarily restricted to the human species and are not present as such in mouse, although moderate (77%) identity exists between Exon C-related sequences in these species. These “human-specific” exons were identified by Sarret et al. (2010) who showed that the splice variants which incorporate these exons are mainly expressed in neurons, as early as fetal development. Using 6.2hPLP(+)Z/FL mice as a model system, we show that while the splice variants from the transgene can be detected in brain through non-quantitative RT-PCR analysis (Figure 3), the archetypal (classic) transcript is expressed to significantly higher levels, as determined by RT-qPCR analysis (Figure 4). Moreover, only the Exon C-containing variant was measurable in the RT-qPCR analysis throughout development; a slight amount of expression of the A′-specific variant was detected at E14.5. As mentioned earlier, it is unknown whether the native *hPLP1* transcripts that incorporate Exon C get translated into protein products. The longest open reading frame is predicted to produce a peptide that corresponds to the last 72 amino acids (residues 205–276) of PLP (Sarret et al., 2010). Nevertheless, Exon C-containing splice variants from 6.2hPLP(+)Z/FL are not expected to contribute to the overall level of β-gal activity due to the introduction of multiple in-frame stop codons downstream from the classic translation start site near the end of *hPLP1* Exon 1.

As noted in Table 1, a residual amount of expression is still present in brain when the wmN1 region is removed from the parental 6.2hPLP(+)Z/FL line, as evidenced by marginally higher levels of β-gal activity in 6.2hPLPΔwmN1 mice over wild-type littermates. In fact, the level was slightly higher at P2 compared to P21. However, removal of the majority of *hPLP1* intron 1 from the parental line resulted in a complete loss of activity (see 6.2hPLPΔ214-8467 in Table 1). These results, coupled with a lack of A or A′-specific variants by 6.2hPLP(+)Z/FL at either P2 or P21, suggest that the residual activity remaining in 6.2hPLPΔwmN1 is not due to expression of “human-specific” splice variants. Thus, the lack of expression by 6.2hPLPΔ214-8467 is likely due to loss of another regulatory element, besides wmN1. Interestingly, Tuason et al. (2008) identified a region just downstream of wmN1, called wmN2, which has weak enhancer activity in brain. Thus, it is possible that the combined loss of these enhancer regions in 6.2hPLPΔ214-8467 accounts for its loss of activity.

Curiously we noticed that expression of the 6.2.hPLP(+)Z/FL transgene was wide-ranging in brain at P2 for Line 777 (Figures 1B, 2), while mice containing the related mPLP(+)Z transgene show far less expression at this age, which is primarily limited to olfactory bulb (Figure 1B). Substantial levels of 6.2hPLP(+)Z/FL expression in brain were also observed at P2 with other transgenic lines (Hamdan et al., 2018), indicating that the early postnatal expression is not due to a peculiarity (such as a position effect) with Line 777. Because 6.2hPLP(+)Z/FL contains a greater amount of 5′-flanking *PLP1* DNA than does mPLP(+)Z, we reasoned that the 6.2hPLP(+)Z/FL transgene may possess a transcription regulatory element important for early postnatal expression, which was not included in the mPLP(+)Z transgene. Therefore, we generated additional transgenic mouse lines that harbor the 2.7hPLP(+)Z transgene, which contains just the orthologous *hPLP1* sequence to that present in mPLP(+)Z, to test whether this affects early postnatal expression in brain. Seven lines (Lines 217, 226, 227, 229, 236, 559, and 563) were established that express the transgene (Table 2). With the exception of Line 226, which is a very low expressing line, expression in brain was higher at P21 than P2 (Table 3). Similar results were obtained by RT-qPCR analysis, which showed that higher levels of transgene RNA are present in brain at P21 than P2 (Table 3). Taken together, these results suggest that a transcription regulatory element is present in *hPLP1* 5′-flanking DNA, located 2.7 to 6.2 kb upstream of Exon 1, which is important for governing early postnatal expression in brain. Where, within this 3.5-kb stretch of DNA, the regulatory element lies remains to be determined. However, a clue to its location may come from the enhancer-trap study by Tuason et al. (2008). Besides the wmN1 and wmN2 enhancer regions, the authors identified a 1,938-bp sequence of *mPlp1* 5′-flanking DNA (called 4,250-Opo) that possesses enhancer-like activity. It is located −4,170 to −2,232 bp upstream of the transcription start site. Hence, the majority of this sequence lies immediately upstream from the 5′-end of the mPLP(+)Z transgene, as does the orthologous *hPLP1* sequence, with respect to the 2.7hPLP(+)Z transgene.

## Data availability statement

The raw data supporting the conclusions of this article will be made available by the authors, without undue reservation.

## Ethics statement

The animal study was reviewed and approved by the Institutional Animal Care and Use Committee at the University of Arkansas for Medical Sciences.

## Author contributions

PP and PW designed the study. PP, DF, and HH performed the experiments. PP and PW wrote the manuscript, with journalistic input from DF and HH. All authors read and approved the submitted version.
