# Supplementary figures and images for: PLP1-lacZ transgenic mice reveal that splice variants containing “human-specific” exons are relatively minor in comparison to the archetypal transcript and that an upstream regulatory element bolsters expression during early postnatal brain development
Source: Front Cell Neurosci. 2023 Jan 11;16:1087145. doi: 10.3389/fncel.2022.1087145 (PMC9875078; doi:10.3389/fncel.2022.1087145)

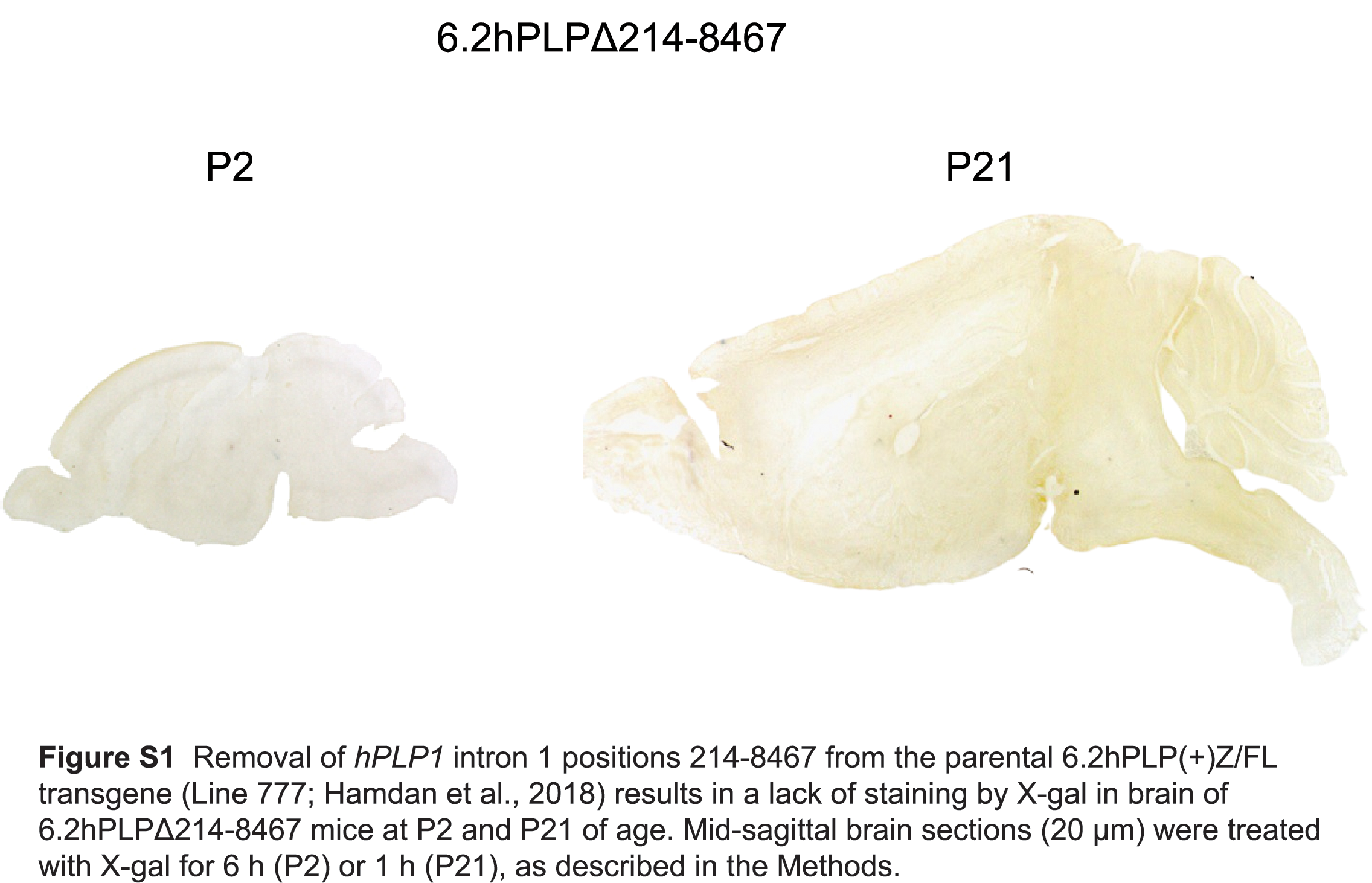

Supplement: Supplementary file 1 [file Image_1.TIF]
